# Health literacy and hypertension-related multimorbidity: unravelling the mediating role of self-management - insights from the lifelines cohort study

**DOI:** 10.1186/s12889-025-22798-x

**Published:** 2025-04-24

**Authors:** L. Loreto, F.G. Linares-Jimenez, J. de Zeeuw, A.F. de Winter

**Affiliations:** 1 Department of Health Sciences, University Medical Center Groningen, University of Groningen, Groningen, The Netherlands; 2 Department of Health Sciences, Global Health Unit, University Medical Center Groningen, University of Groningen, Groningen, The Netherlands

**Keywords:** Health literacy, Hypertension, Multimorbidity, Self-management, Mediation

## Abstract

**Background:**

People with limited health literacy and hypertension may lack the self-management skills needed to manage their illness, contributing to the onset and progression of important adverse health outcomes (e.g., multimorbidity). This study aimed to assess the association between health literacy and the onset and progression of hypertension-related multimorbidity among people with hypertension and whether this association is mediated by components of self-management (i.e., motivation, self-efficacy beliefs and problem-solving).

**Methods:**

The study sample included data from 21,725 adult participants with hypertension who were followed in the Lifelines Cohort Study. Using causal mediation analysis with natural effect models, the total and direct effect of health literacy on hypertension-related multimorbidity and the indirect effects of motivation, self-efficacy beliefs and problem-solving were studied.

**Results:**

First, after controlling for age, sex, smoking status, education level and monthly income, the total and direct effects of health literacy on the onset of hypertension-related multimorbidity were null (OR: 1.00, 95% CI: 0.99–1.01); however, an increase in problem-solving scores had an indirect effect on the onset of hypertension-related multimorbidity (OR: 0.99, 95% CI: 0.98–0.99). Second, a change from limited health literacy to adequate health literacy (OR: 0.99, 95% CI: 0.98–0.99) and an increase in motivation (OR: 0.99; 95% CI: 0.99–1.00), self-efficacy beliefs (OR: 0.99; 95% CI: 0.98–0.99) and problem-solving (OR: 0.99; 95% CI: 0.98–0.99) scores had direct and indirect effects, respectively, on the progression of hypertension-related multimorbidity.

**Conclusions:**

A change from limited health literacy to adequate health literacy had no direct effect on the onset of hypertension-related multimorbidity but did decrease the likelihood of its progression. Problem-solving indirectly mediated only the onset; meanwhile, motivation, self-efficacy beliefs and problem-solving mediated the progression of hypertension-related multimorbidity. Improving health literacy and self-management skills can play a crucial role in preventing or delaying the onset or progression of hypertension-related multimorbidity. These improvements can significantly reduce the disease burden for individuals with limited health literacy and those living with hypertension-related multimorbidity.

**Supplementary Information:**

The online version contains supplementary material available at 10.1186/s12889-025-22798-x.

## Background

Limited health literacy (LHL) is a significant public health challenge [1] because it is associated with different chronic diseases and might serve as a potential precursor to multimorbidity [2]. Health literacy is defined as the degree to which people can obtain, read, understand and use health information and services to promote and maintain good health [3]. Consequently, LHL is understood as the degree of health literacy where the capacity to effectively handle one’s health is hindered, usually due to the underdevelopment or lack of health literacy skills [4]. Patients with LHL and a chronic illness, such as hypertension, might find it challenging to conduct simple yet essential illness-related tasks, such as following a prescription or interpreting physical measurements such as blood pressure [5]. LHL affects nearly half of the European population, mainly among people from ethnic minorities, people with low income, those with lower educational attainment and older age [6]. LHL is linked to decreased quality of life, increased morbidity and mortality, increased healthcare resource utilization, and increased healthcare costs [5, 7].

Hypertension has a global prevalence of approximately 32% [8] and stands out as a key risk factor for multimorbidity [9, 10]. Studies have revealed that approximately 8 out of 10 people with hypertension live with multimorbidity, the latter being the co-occurrence of two or more chronic illnesses [9]. Multimorbidity in people with hypertension is recognized as cardiometabolic multimorbidity (e.g., cardiovascular disease, type 2 diabetes and chronic kidney disease) [10, 11] and non-cardiometabolic multimorbidity (e.g., psychiatric or respiratory illnesses) [11, 12]. The burden of illness among people living with multimorbidity mirrors patterns observed in those living with LHL [13]. Given the care complexity of people living with multimorbidity, limiting multimorbidity has acquired substantial relevance for researchers, healthcare providers and policymakers [2, 11]. Multimorbidity in the general population [14, 15], and in patients with chronic kidney disease [2, 16] is more prevalent among people with LHL. However, the association between the onset and progression of multimorbidity and health literacy, and the potential underlying mechanisms explaining such association remain unclear [2]. For the purposes of the present study, the co-occurrence of one or more chronic illnesses alongside hypertension will be referred to as hypertension-related multimorbidity (HRM) [10, 11]. Likewise, the first appearance of HRM will be referred to as the onset of HRM, while the occurrence of more chronic illnesses after the first appearance (not necessarily reflecting the order of the illness’s appearance) will be referred to as the progression of HRM.

Potential mechanisms through which LHL might explain the onset and progression of HRM include the underdevelopment of self-management skills [7, 14, 16]. Self-management in chronic illness is the ability to overcome changes related to the management of such illness and has been described in different self-management models including the health belief model [17], the social cognitive model and the theory of planned behaviour [7]. In line with these models and the health literacy framework proposed by Paasche-Orlow and Wolf [18], three relevant self-management skills have been highlighted: motivation, self-efficacy beliefs and problem-solving. Motivation is defined as the will to improve health, self-efficacy beliefs referred to as the self-confidence to implement specific actions that support health, and problem-solving to the capacity to overcome illness and treatment limitations. These skills, while independent are reflected together in daily behaviours, such as attending medical appointments, self-monitoring blood pressure and developing emergency action plans [19]. In this sense, a patient with adequate health literacy (AHL) will have more robust self-management skills to adopt the wide set of behaviours needed to manage hypertension and ultimately will prevent or delay the onset and progression of HRM [7, 20]. Current research has identified that self-management might explain the development of adverse health-related outcomes [7, 16, 21]. However, although hypothesized, the role of self-management skills as potential underlying mechanisms in the association between health literacy and HRM remains unclear.

Disentangling the role of self-management skills will allow healthcare professionals to individualize healthcare support according to the unique needs of each patient [22]. As such, this study examined the associations between health literacy and the onset and progression of HRM and whether these associations were mediated by components of self-management skills such as motivation, self-efficacy beliefs and problem-solving.

## Methods

### Sample and procedures

The study sample was derived from the first and second assessments of the Lifelines Cohort Study. Lifelines is a multi-disciplinary prospective population-based cohort study examining in a unique three-generation design the health and health-related behaviours of 167,729 persons living in the North of The Netherlands. It employs a broad range of investigative procedures in assessing the biomedical, socio-demographic, behavioural, physical and psychological factors, which contribute to the health and disease of the general population, with a special focus on multi-morbidity and complex genetics [23, 24]. Baseline collection (T1) was performed between 2006 and 2013 for 167,729 participants, the second assessment (T2) between 2014 and 2019 for 165,100 participants, and the third assessment (T3) between 2019 and 2023 [23, 25]. Prior to participation in the study, each participant provided written informed consent. The Lifelines Study was conducted following the principles of the Declaration of Helsinki and the research codes of the University Medical Center Groningen.

**Fig. 1:**
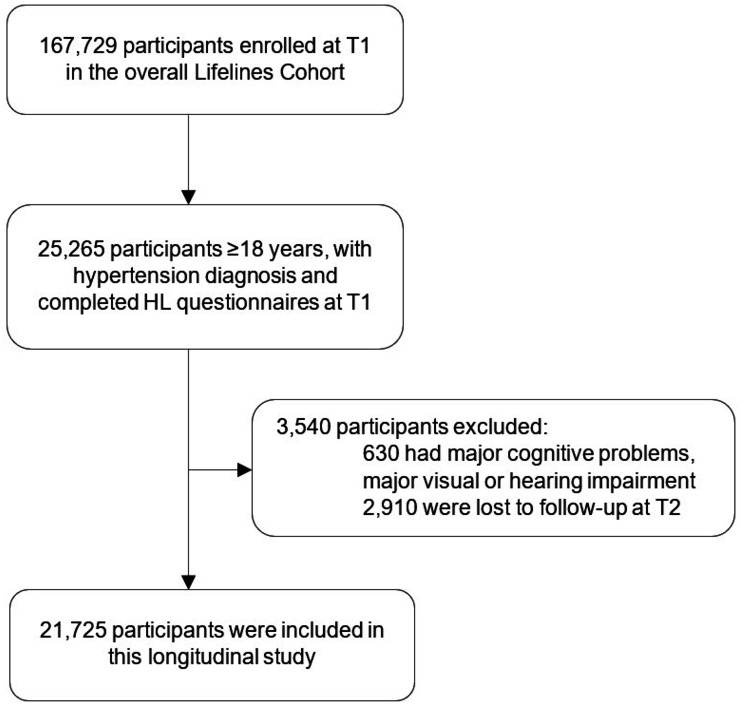
Patient selection flow

Participants were eligible for our study if they were older than 18 years, had been diagnosed with hypertension and had completed health literacy questionnaires. Hypertension was defined as persistently high blood pressure (≥ 140/90 mmHg) [26]; however, to allow for a more comprehensive construct, participants were required to meet at least one of the following criteria at T1: (1) self-reported diagnosis of hypertension; (2) measured diastolic blood pressure ≥ 140 mmHg and/or systolic blood pressure ≥ 90 mmHg; or (3) use of drugs to manage hypertension (i.e., Anatomical Therapeutic Chemical (ATC) codes C02, C03, C07, C08 and C09). Exclusion criteria included the presence of major cognitive impairments (i.e., diagnosis of dementia, Mini-Mental Status Exam (MMSE) score < 24), major visual or hearing impairments, or loss to follow-up at T2. The final sample for this longitudinal study included 21,725 participants (Fig. 1).

### Measurements

#### Health literacy

Health literacy was measured at T1 with three validated items from Chew et al. [27]: (1) How often do you have trouble understanding your medical situation because you have difficulty with the written information? (2) How sure are you of yourself when you fill out medical forms? (3) How often does someone help you with reading information materials from the hospital or another healthcare provider?

The participants answered the questions on a Likert scale ranging from 1 (never) to 5 (always). The scores from the first and third questions were reversed, and then all the scores were summed, resulting in a scale ranging from 3 to 15. The final score was dichotomized into LHL (≤ 12 points) and AHL (≥ 13 points). This cut-off point has been used in previous studies with the same database [1, 2, 16].

### Composition of individual illness and illness domains

In accordance with previous studies and the 10th edition of the International Statistical Classification of Diseases and Related Health Problems (i.e., ICD-10), 34 individual illnesses were composed of self-reported clinical information and measurements (e.g., electrocardiogram reports, spirometry and laboratory results) [2]. The use of subjective and objective variables was considered to increase illness classification accuracy. Individual illnesses were used to compose 10 illness domains at T1 and T2, namely, endocrinology, cardiovascular, haematology, renal, respiratory, dermatology, psychiatry, gastrointestinal, neurological, and musculoskeletal domains (see Additional file 1). An illness domain was considered to be affected if at least one individual illness was present. The illness domains were then used to calculate an illness score (0–10) based on the number of illness domains affected at each assessment.

Special considerations were applied to some illness domains at T2. First, source variables for the dermatological domain at T2 were missing; therefore, source variables from T3 were used instead to prevent missing data. Notably, the time difference between T2 and T3 was 1–3 years; thus, we expected similar trends among the variables. Second, two source variables (i.e., *serum creatinine* and *albuminuria*) were used to determine the renal illness domain. The percentages of missing data for the *albuminuria* variable at T1 and T2 were 55% and 97%, respectively. Although the percentages of missing data for the *albuminuria* variable were high, this variable allowed us to detect participants with milder stages of chronic kidney disease; thus, they were included in the final analyses.

### Onset and progression of hypertension-related multimorbidity

First, the presence of HRM at baseline was calculated and defined when at least one other illness domain was affected simultaneously with hypertension at T1 (i.e., baseline illness score > 1). Second, the onset and progression of HRM were calculated as the prevalence of HRM at T1 and the cumulative incidence of HRM at T2. The onset of HRM was present in those participants with no HRM at baseline who then had at least one other illness domain affected at T2 (i.e., illness score > 1). Moreover, progression of HRM was defined in participants with the presence of HRM at baseline who had at least one additional illness domain affected at T2 (i.e., T2 illness score > T1 illness score).

### Motivation, self-efficacy beliefs and problem-solving

Motivation was assessed at T1 with two 5-item subscales of the validated 30-item Self-Management Ability Scale (SMAS-30), namely, (1) taking initiative (e.g., *How often do you take the initiative to actively engage in something?*), and (2) investment behaviour (e.g., *Do you make sure you exercise enough to stay fit longer?).* The participants answered the items of both subscales on a 6-point Likert scale from 1 (never) to 6 (very often), resulting in 2 subscores ranging from 0 to 100, with higher scores indicating greater motivation. These subscores were averaged into a single motivation score [28, 29].

Self-efficacy beliefs were also assessed at T1 with a 5-item subscale of the SMAS-30 (e.g., *“Do you succeed in taking good care of yourself?”)*. The participants answered the items on a 5-point Likert scale from 1 (definitely not) to 5 (definitely yes), resulting in a score ranging from 0 to 10, with higher scores indicating greater self-efficacy beliefs [28, 29].

Problem-solving was assessed at T1 with the 10-item Goal Adjustment Scale (GAS), which is based on the goal adjustment theory. The GAS is distributed into two subscales concerning (1) goal disengagement (4 items, e.g., “*It’s easy for me to reduce my effort towards the goal*”) and (2) goal engagement (6 items, e.g., “*I think about other new goals to pursue”*). The participants answered all statements on a 5-point Likert scale ranging from 1 (disagree) to 5 (agree). Two scores from the goal disengagement subscale were reversed, and then all subscores were averaged, resulting in a final score ranging from 10 to 50, with higher scores indicating higher problem-solving skills [30].

### Other variables

Covariates were assessed at T1 with self-reported information and included age, sex assigned at birth, educational level, monthly household income and smoking status. Educational level was measured on an 8-item ordinal scale and categorized as low (no education or primary education), intermediate (secondary education) or high (higher vocational or university education) based on an expert assessment of the Dutch educational system [31]. Monthly household income was assessed with an 8-item ordinal scale and classified by our team into three categories: <1000 euros, 1000–3000 euros and > 3000 euros, fitting the available data to the low-income threshold (990 EUR per month) and wealth distribution reported by the Central Bureau for Statistics of the Netherlands at the time this measurement occurred [32]. Participants were considered smokers if they self-reported smoking in the previous month [16].

### Analysis

First, we calculated descriptive statistics to evaluate differences between the participants with LHL and those with AHL. Chi-square tests and independent sample t-tests were used for categorical and continuous variables, respectively. Approximately 6.6% of the total sample data were missing at random (missing intervals from variables between 0.6% and 97%). Multiple imputation by chained equations (*n* = 20) was applied with the *mice* (v3.15.0) package for R (v4.2.0). Predictor variables with a large minimum marginal R-square (*r* = 0.02) were selected, and variables that showed collinearity were excluded from the predictor matrix [33]. To avoid missing data, outcome variables (onset and progression of HRM) were composed of multiple imputed data [34]. The validity of the imputations was determined, and further analyses were conducted with multiple imputed data.

**Fig. 2:**
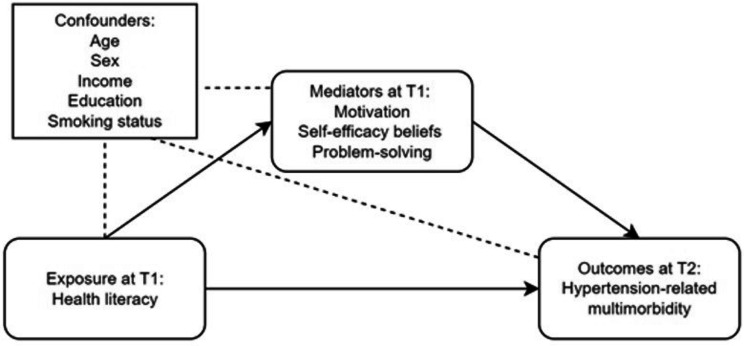
Causal mediation model

Second, we assessed the mediating role of motivation, self-efficacy beliefs and problem-solving with causal mediation (see Fig. 2) using natural effect models with the *medflex* (v0.6-7) package for R (v4.2.0). Causal mediation analysis is based on the counterfactual framework and enabled us to denote the potential outcome that would have been observed when the mediator value remains as the naturally observed i.e., the direct effect, as well as to observe the expected outcome when the mediator value had changed to the value it would have taken if unexposed, i.e., the indirect effect [35]. To allow the presence of different exposures and mediators, the original dataset was expanded using an imputation approach. Three natural effect models (one for each mediator) were fitted for both outcome variables, where AHL was considered the exposure variable (AHL = 1). The model parameter estimates were exponentiated to calculate natural effect odds ratios and confidence intervals. Only the self-efficacy beliefs mediator violated the exposure-mediator independence assumption, for which an additional multivariable model that included all three mediators was fitted. The mediation models were adjusted for age, sex, educational level, monthly income and smoking status and were considered statistically significant if *p* < 0.05. Finally, we conducted sensitivity analyses without the variable *albuminuria* for the renal illness domain at both time points. All data preparation and analyses for the present project were conducted with R (v4.2.0) for Windows.

## Results

### Participant characteristics

Among the 21,725 included participants, approximately one-third (31.1%) reported LHL (see Table 1). More than half of our total sample was women (59.4%), with a mean age of 52.4 years (SD = 11.6), middle monthly income and middle educational level. Among the proportion of participants with LHL (31.1%), significantly lower motivation, self-efficacy beliefs and problem-solving score mean (59.9, 75.4 and 32.8, respectively) were observed, along with a significantly higher HRM at baseline (72.7%) than those with AHL. Similarly, participants with LHL had a higher onset of HRM (7.4%) and progression of HRM (5.8%) at T2 than did participants with AHL (8.3% and 4.3%, respectively). The number of illness domains that accumulated was between 3 and 10, and participants with LHL were more likely to have a greater number of affected illness domains.

**Table 1:**
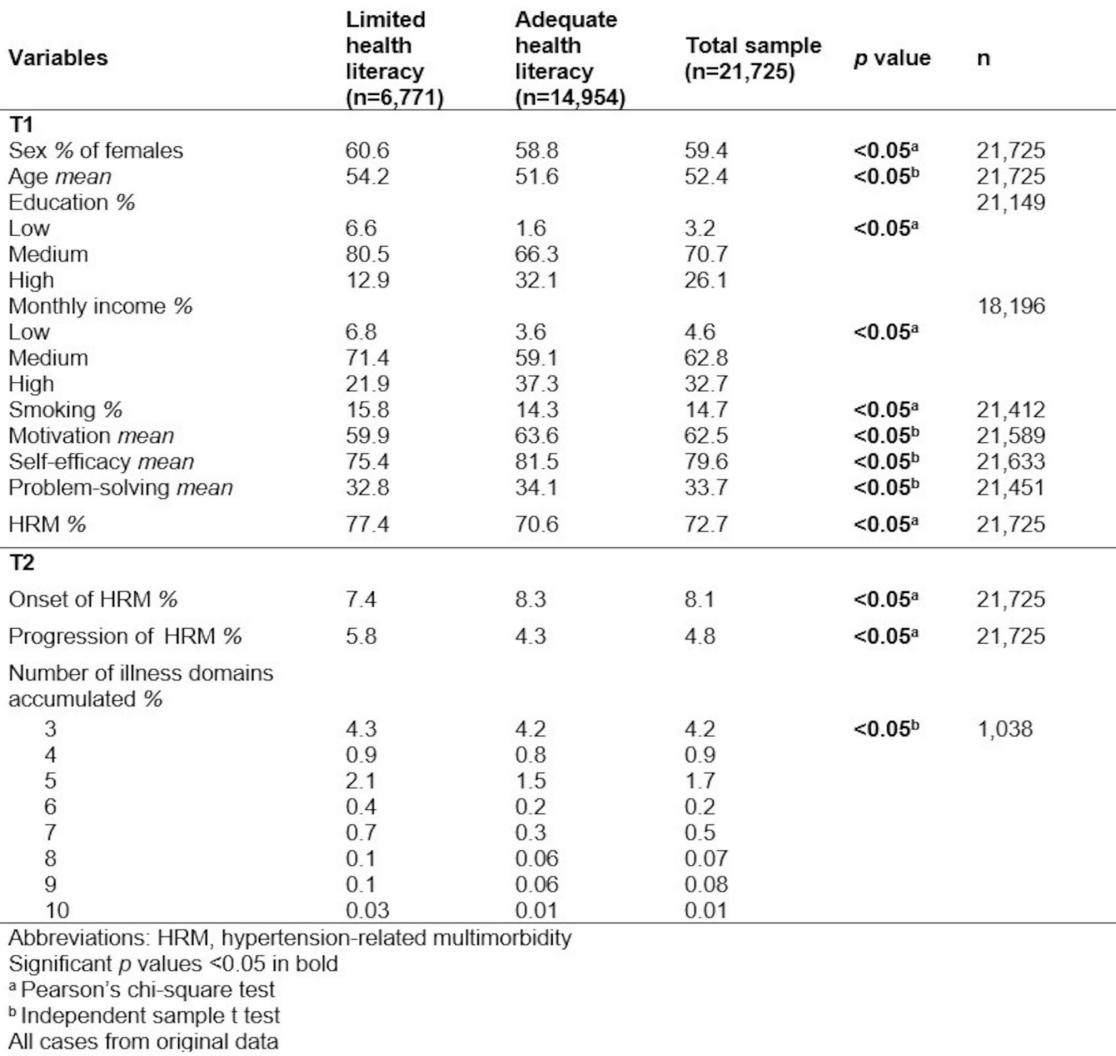
Comparison of T1 characteristics and T2 outcomes of people with hypertension, and limited or adequate health literacy

The participants with LHL had a higher HRM prevalence and HRM incidence in all illness domains, except for the dermatological domain (see Table 2). At baseline, the illness domains with the highest prevalence of affection in the LHL group were the endocrinology (67%), gastrointestinal (60.3%), respiratory (25.3%), and cardiovascular (19%) domains. Moreover, higher cumulative incidence rates of affection were observed in the musculoskeletal (15.6%) and psychiatric (8.1%) illness domains.

**Table 2:**
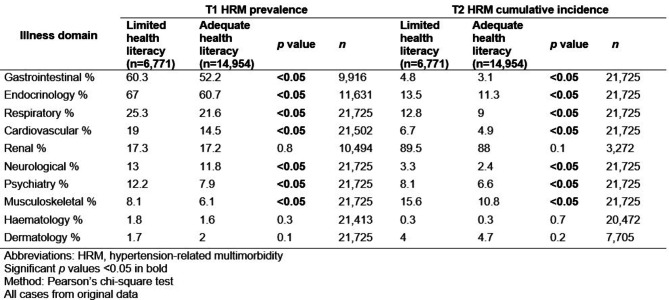
Comparison of the T1 prevalence and T2 cumulative incidence of affected illness domains between people with hypertension and those with limited or adequate health literacy

### Effect of health literacy and self-management skills

The estimates of the total effect (TE) and natural direct effect (NDE) (i.e., the effect of the exposure on the outcome without the mediator) revealed that a hypothetical transition from LHL to AHL had no effect on the odds of the onset of HRM (TE / NDE - OR: 1.00, 95% CI; 0.99–1.01) (see Table 3). However, the natural indirect effect (NIE) estimates (i.e., the effect of the exposure on the outcome through the mediator) suggested a significant mediating role of problem-solving on the onset of HRM (OR: 0.99, 95% CI: 0.98–0.99). Specifically, an increase in problem-solving scores among participants with LHL to scores equivalent to those hypothetically observed in participants with AHL decreased the odds of the onset of HRM.

**Table 3:**
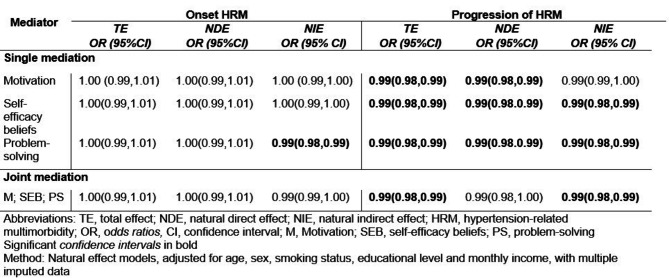
Total effect, natural direct effect and natural indirect effect odds ratios of motivation, self-efficacy beliefs and problem-solving, on the association between health literacy and onset and progression of hypertension-related multimorbidity

In contrast, TE and NDE estimates (TE / NDE - OR: 0.99, 95% CI: 0.98–0.99) showed that the odds of the progression of HRM significantly decreased when hypothetically transitioning from LHL to AHL. NIE estimates suggest that an increase in separate self-management skills decreased the odds of the progression of HRM, with two out of three self-management skills (self-efficacy beliefs and problem-solving) showing significant mediation effects (NIE - OR: 0.99, 95% CI: 0.98–0.99).

Finally, NIE estimates from the joint model, suggest that an increase in all three self-management skills decreased the odds of both the onset of HRM (OR: 0.99, 95% CI: 0.99–1.00) and the progression of HRM (OR: 0.99, 95% CI: 0.98–0.99). TE, NDE, and NIE estimates of LHL on the onset and progression of HRM remained similar in the sensitivity analysis (see Additional file 2).

## Discussion

We studied the association between health literacy and the onset and progression of HRM and the extent to which this association was mediated by components of self-management skills such as motivation, self-efficacy beliefs and problem-solving. We found that after adjusting for age, sex, monthly income, educational level and smoking status, a change from LHL to AHL did not have a NDE on the onset of HRM; however, it significantly decreased the likelihood of the progression of HRM. Moreover, only a higher score of problem-solving skills had a statistically significant mediating effect on the onset of HRM. While, all three self-management skills, i.e., motivation, self-efficacy beliefs and problem-solving skills, mediated the progression of HRM, with significant effects found on self-efficacy beliefs and problem-solving. To the best of our knowledge, this is the first study to assess this association and mediating pathway.

### Health literacy and HRM

Our causal mediation analysis revealed that a change from LHL to AHL had a null TE and NDE on the onset of HRM. However, a decrease in the progression of HRM was observed. These different results may be explained by two factors. The first is a methodological explanation, in which the sample size of the group with no HRM at baseline (27.3%) was smaller than that of the group with the presence of HRM at baseline (72.7%). A smaller sample size might have prevented our findings from reaching enough effect and significance levels. The lower prevalence of hypertension with no HRM at baseline might be explained by the fact that around half of the adults with hypertension are unaware of their illness, making the presence of HRM at baseline before diagnosis more likely to occur [26, 36]. The second one is causal, as it might be that participants with no HRM experienced less illness burden, reflecting potentially better self-management skills. In this sense, managing only one illness (i.e., hypertension) might be simpler than managing multiple illnesses (i.e., HRM) at the same time [10]. Our mixed findings are supported by previous studies showing that no relationship was found between health literacy and multimorbidity after controlling for covariates among people with at least one chronic illness [37]. In contrast, more recent studies have reported a positive association between health literacy and multimorbidity among different populations, such as people with chronic kidney disease and cardiovascular disease [2, 16] and the general population [14, 15, 38].

Interestingly, our study found both high NDE and NIE on the progression of HRM. According to the model of Paasche-Orlow and Wolf [18], the high NDE may suggest that additional underlying mechanisms exist to explain the relationship between health literacy and the progression of HRM. Additional underlying mechanisms include the access and utilization of healthcare, and strong communication with healthcare providers, family, and friends. Whereas, the high NIE at the same time highlights the important role of self-management in HRM, specifically but not limited to the progression of HRM.

### Self-management and HRM

In accordance with our findings, motivation, self-efficacy beliefs and problem-solving skills are important components of the onset and progression of HRM. Our findings revealed a mediating effect of motivation on both the onset and progression of HRM. This mediating role reached significance levels in our joint model but not in the single mediation model, which might be explained by two reasons in line with Coventry et al. (2014). First, depression might strongly influence the effect of motivation [39]. However, for our study, depression was considered part of HRM; therefore, no adjustment was possible. Second, the adjustment for monthly income and educational level might have hindered the effect of motivation on HRM, given the close relationship between them. The qualitative study of Coventry et al. (2014) described the relevance of motivation from practitioners’ and people’s perspectives. Practitioners, for example, stated that motivation alongside depression are important barriers to self-management engagement. In addition, people might experience low motivation levels due to loneliness and the absence of social support, alongside financial problems [39]. Motivation appears to be a mainstay of the self-management concept and should be further studied.

Self-efficacy beliefs mediated only the association between health literacy and the progression of HRM. People with higher levels of self-efficacy beliefs, irrespective of their health literacy level, are more likely to follow their medical treatment. Therefore, the likelihood of the progression of HRM is expected to be lower. Our findings are supported by Stock et al. (2021), who reported a negative association between self-efficacy scores and the number of chronic illnesses in people from the general population [40]. Similarly, a study conducted by Xie et al. (2020) revealed a mediating effect of self-efficacy between older age and adherence to diet therapy in people with hypertension and diabetes [41]. These results, in addition to our findings, might suggest that self-efficacy has an important mediating effect on social determinants of health, such as health literacy, and health outcomes related to chronic illness. Nevertheless, further research is needed, specifically in our target population.

Finally, problem-solving skills mediated the associations between health literacy and both the onset and progression of HRM. In this sense, participants with LHL but adequate levels of problem-solving skills had a lower likelihood of the onset and progression of HRM. People with hypertension need to integrate complex and daily medical treatment, including medication adherence, lifestyle changes, and self-monitoring of blood pressure. Thus, it is essential to first recognize treatment barriers and then implement effective solutions to overcome those barriers. Currently, growing literature is emerging, although with mixed results, concerning the relevance that problem-solving skills have for the management of chronic illnesses. A systematic review revealed that problem-solving interventions in people with diabetes lead to better illness control rates [42]. In contrast, a randomized control trial that assessed a problem-solving intervention in people with chronic obstructive pulmonary disease did not show any effect compared with standard care [43]. These findings might be translated to different chronic illnesses, such as HRM. However, the current literature is inconclusive, and the role of problem-solving in HRM needs to be further explored.

### Strengths and limitations

The strengths of our study include the use of a causal mediation analysis over other mediation analyses (e.g., structural equation modelling) which allowed us to better understand the potential causal role of the different components of self-management behind the onset and progression of HRM in different hypothetical scenarios (e.g. LHL vs. AHL). Additionally, our study included a large population-based sample of participants, with a longitudinal design and a comprehensive approach for hypertension and HRM classification. Furthermore, our study included the measurement of both the onset and progression of HRM, which has allowed us to increase the understanding of HRM aetiology [44]. Moreover, the tools we used to measure self-management skills were both theory-based and validated.

Limitations of our study should also be considered. First, participants with LHL might have found it challenging to answer the health literacy questionnaires. This factor might have led to participants not answering, and thus, to an underestimation of participants with LHL. Second, data on albuminuria were missing from our sample in 55% and 97% of participants at T1 and T2, respectively, which could have selection bias implications. Notably, missing data were missing at random, and its missingness was related to logistical reasons of the Lifelines Study. Thus, with the use of the multiple imputation approach, it is unlikely that this approach might lead to important bias. Third, previous research has established a potentially relevant role of ethnicity in multimorbidity in the general population [44]. However, given the study design of the Lifelines Study, adjustment for this important covariate was not possible. Therefore, the generalizability of our study might be limited to populations similar to those of the Lifelines Study.

### Implications

Our study showed that while LHL had direct negative effects on the progression of HRM, an increase in self-management skills, especially problem-solving skills, might help mitigate such effects. Our findings suggest that integrating a comprehensive healthcare model where not only health literacy is addressed but also the development and enhancement of problem-solving skills is targeted may reduce the negative consequences of HRM. Healthcare professionals can benefit from receiving educational programs and training to increase the awareness of the relevance that health literacy and self-management skills have for the onset and progression of HRM [42, 43]. Moreover, people could benefit from this, as subsequently, there could be an improvement in clinician-patient communication. For example, healthcare providers could make use of strategies such as “teach-back” and written and visual materials to complement verbal communication [45] while guiding their patients in important self-management skills such as blood pressure tracking and developing a contingency plan for medical emergencies. Supporting patients in enhancing their health literacy and self-management skills will not only help them manage their illness but also empower them, ultimately resulting in a reduction in the illness burden.

Future research should continue to unravel potential underlying mechanisms between health literacy and the onset and progression of HRM. Furthermore, understanding the illnesses’ trajectories might help to identify and understand the prognosis of people at the highest risk to prevent the onset and progression of HRM. Therefore, the study of the pattern of the progression of HRM should also be considered [44]. Ideally, research should continue with longitudinal designs, with heterogeneous populations that could increase the generalizability of research and make use of instruments that measure self-management skills in the illness context.

## Conclusion

This study revealed that a change from LHL to AHL in a group of people with hypertension had no effect on the onset of HRM but did decrease the likelihood of the progression of HRM. Self-management skills play a relevant role in the onset and progression of HRM. Problem-solving skills mediated the association between health literacy and the onset of HRM. Moreover, motivation, self-efficacy beliefs and problem-solving skills mediated the progression of HRM. Improving LHL and problem-solving skills in people with hypertension might prevent or delay the onset and progression of HRM.

## Electronic supplementary material

Below is the link to the electronic supplementary material.

Supplementary Material 1

Supplementary Material 2

## Data Availability

Data may be obtained from a third party and are not publicly available. Researchers can apply to use the Lifelines data used in this study. More information about how to request Lifelines data and the conditions of use can be found on their website (https://www.lifelines-biobank.com/researchers/working-with-us).

## Abbreviations

AHL: Adequate Health Literacy
GAS: Goal Adjustment Scale
HRM: Hypertension-related Multimorbidity
LHL: Limited Health Literacy
SMAS-30: Self-Management Ability Scale
