# Supplementary material for: Health literacy and hypertension-related multimorbidity: unravelling the mediating role of self-management - insights from the lifelines cohort study
Source: BMC Public Health. 2025 Apr 24;25:1530. doi: 10.1186/s12889-025-22798-x (PMC12020009; doi:10.1186/s12889-025-22798-x)
Supplement: Supplementary file 1 — Supplementary Material 1 [file 12889_2025_22798_MOESM1_ESM.docx]

**Additional file 1 - Diagnosis criteria**

1. Endocrinology domain affected, if at least one of the following single illnesses were present:
   1. Diabetes (IV-E10/E11/E12/E14)
      1. Self-reported diabetes (type 1 and type 2), or
      2. use of oral anti-diabetics (ATC A10B) and/or insulin (ATC A10A, or
      3. fasting glucose levels >=6.99, or
      4. non-fasting glucose level >=11.0,or
      5. HbA1c level>=6.5
   2. Hypothyroidism (IV-E03)
      1. Use of thyroid hormones (ATC H03), or
      2. TSH>4 & FT4<11.0, or
      3. TSH>10
   3. Hyperthyroidism (IV-E05)
      1. TSH <0.5 & FT4>19.5
   4. Hypercholesterolemia (IV-E78.0)
      1. Self-reported myocardial infarction and cholesterol >=5.0, or
      2. Use of lipid lowering medication (ATC C10A, C10B), or
      3. Cholesterol >= 6.5 mmol/L
2. Cardiovascular domain affected, if at least one of the following single illnesses were present:
   1. Vascular disease (IX-I21, IX-I25.2, IX-I64)
      1. Self-reported myocardial infarction, stroke, aneurysm, angioplasty/bypass presence, stenosis, atherosclerosis, heart valve presence and
      2. use of vitamin K antagonists (ATC B01AA) or acetylsalicylic acid or clopidogrel (ATC B01AC) or simvastatin (ATC C10AA), or
      3. myocardial infarction on ECG
   2. Heart failure (IX-I50)
      1. Self-reported heart failure, and
      2. use of (any) heart failure-related medication (diuretics, ACE-I, ANG II antagonists, aldosterone antagonist, beta-blocker, calcium antagonist) (ATC C03,C09,C07,C08)
   3. Atrial fibrillation (AF) (IX-I48)
      1. Self-reported AF, self-reported AF-diagnosed by MD, and
      2. use of vitamin K antagonists (ATC B01AA), or
      3. AF on ECG
   4. Pacemaker (XXI-Z95.0)
      1. Self-reported
   5. Heart transplant (XXI-Z94.1)
      1. Self-reported
3. Haematology domain affected, if at least one of the following single illnesses were present:
   1. Anaemia (III-D50D64)
      1. Self-reported anaemia and use of iron supplementation (ATC B03), or
      2. Hb <6.0 if female, or
      3. Hb<6.5 if male.
   2. Thrombotic disease
      1. Self-reported thrombosis (IX-I82) and use of anti-thrombotic drugs (ATC B01AA), or
      2. self-reported lung emboli (IX-I26) and use of anti-thrombotic drugs (ATC B01AA), or
      3. self-reported coagulation disorder (III-D68.9) and use of anti-thrombotic drugs (ATC B01AA), or
      4. thrombocytosis (III-D47.3)= Thrombocyte count >750*10E9/L.
   3. Haemorrhagic disease
      1. Self-reported coagulation disorder (III-D68.9) and use of anti-haemorrhagic drugs (ATC B02), or
      2. Thrombocytopenia (III-D69.3/D69.4/D69.4/D69.5/D69.6)=Thrombocyte count <60*10E9/L
4. Renal domain affected if:
   - 1. Estimated glomerular filtration rate (eGFR), calculated with CKD-EPI formula <60 ml/min/1.73m2, or
     2. eGFR>=60 ml/min/1.73m2, and albuminuria assessed by 24 h urine≥30 mg/24 h
5. Respiratory domain affected, if at least one of the following single illnesses were present:
   1. COPD (X-J44)
      1. GOLD classification I-IV (FEV1/FVC <0.7), or
      2. Self-reported COPD & use of respiratory medication (ATC R03), or
      3. Coughing/phlegm production during day or night, or immediately after waking up during at least 3 months per year (Chronic mucus hypersecretion (X-J42), (Chronic bronchitis (X-J40) ), and use of respiratory medication (ATC R03)
   2. Asthma (X-J45)
      1. Self-reported asthma and self-reported-diagnosed by MD, or
      2. Self-reported symptoms of wheezing/ dyspnoea and use respiratory medication (ATC R03), or
      3. Self-reported asthma and use respiratory medication (ATC R03)
   3. Chronic sinusitis (X-J32)
      1. Self-reported sinusitis and use of decongestant medication (ATC R01)
6. Dermatology domain affected, if at least one of the following single illnesses were present:
   1. Eczema (XII-L30.9)
      1. Self-reported eczema and use of emollients and/or dermatological corticosteroids (ATC D02)
   2. Psoriasis (XII-L40.9)
      1. Self-reported psoriasis and psoriasis suppressor (ATC D05)
   3. Severe acne (XII-L70.9)
      1. Self-reported severe acne and use of acne suppressor (ATC D10)
7. Psychiatry domain affected, if at least one of the following single illnesses were present, based on outcomes from MINI interview:
   1. Anxiety disorder (V-F40-F41)
      1. Generalized anxiety disorder, or
      2. Social phobia, or
      3. Panic disorder, or
      4. Use of anxiolytics (ATC N05B)
   2. Depressive disorder (V-F32.0-33.0)
      1. Major depressive disorder, or
      2. Dysthymic disorder and use of antidepressants (ATC N06A)
8. Gastrointestinal domain affected, if at least one of the following single illnesses were present:
   1. Fatty liver disease (alcoholic and non-alcoholic) (XI-K75.8) (XI-K70.0)
      1. HSI>36, where HSI= 8 * (ALT/AST) + BMI (+2 if female) (+2 if diabetes)
   2. Gastric disease (XI-K25)
      1. Self-reported peptic ulcer and use of H2-antagonists, prostaglandins or proton-pump inhibitors (ATC A02B), or
      2. Self-reported gastric symptoms: heartburn, stomach pain, nausea, vomiting, reflux, and use of H2-antagonists, prostaglandins, proton-pump inhibitors (ATC A02B)
   3. Ulcerative Colitis (XI-K51)
      1. Self-reported ulcerative colitis
   4. Crohn’s disease (XI-K50)
      1. Self-reported Crohn’s disease
   5. Celiac Disease (XI-K90)
      1. Self-reported celiac disease, and
      2. Self-reported gluten free diet
   6. Irritable Bowel Syndrome (IBS) (XI-K58)
      1. Self-reported IBS and use of medication for functional gastrointestinal disorders, constipation and antidiarrheals (ATC A03,A06A,A07)
9. Neurological domain affected, if at least one of the following single illnesses were present:
   1. Migraine (VI-G43)
      1. Self-reported migraine and use of migraine medication (ATC N02C)
   2. Back or neck hernia (XIII-51.2)
      1. Self-reported back or neck hernia
   3. Epilepsy (VI-G40)
      1. Self-reported epilepsy and use of anti-epileptics (ATC N03)
   4. Multiple Sclerosis (VI-G35)
      1. Self-reported multiple sclerosis
   5. Parkinson’s disease (VI-G20)
      1. Self-reported Parkinson’s disease and use of anti-Parkinson’s drugs (ATC N04B)
10. Musculoskeletal domain affected, if at least one of the following single illnesses were present:
    1. Rheumatoid Arthritis (XIII-M06.9)
       1. Self-reported rheumatoid arthritis and use of NSAIDS/DMARDS/ TNF-alpha inhibitors/ IL inhibitors/ B- and T-cell inhibitors and/or methotrexate (M01, L04AB, L04AA24, L01XC02, L01BA01)
    2. Self-reported symptoms of pain in hands/feet, or joint stiffness, and use of NSAIDS/DMARDS/ TNF-alpha inhibitors/ IL inhibitors/ B- and T-cell inhibitors and/or methotrexate (M01, L04AB, L04AA24, L01XC02, L01BA01)
    3. Arthrosis (XIII-M19.9)
       1. Self-reported arthrosis and use of analgesics (NSAIDs or opioids) (ATC N02, M01A), or
       2. self-reported symptoms of pain in hands & feet and use of analgesics (NSAIDs or opioids) (ATC N02, M01A), or
       3. self-reported symptoms of joint stiffness and use of analgesics (NSAIDs or opioids) (ATC N02, M01A)
    4. Gout (XIII-M10)
       1. Use of anti-gout medication (ATC M04)
    5. Osteoporosis (XIII-M81)
       1. Self-reported osteoporosis and use of calcium D3 or bisphosphonates (ATC A12AX, M05B)
