# Supplementary material for: Health literacy and hypertension-related multimorbidity: unravelling the mediating role of self-management - insights from the lifelines cohort study
Source: BMC Public Health. 2025 Apr 24;25:1530. doi: 10.1186/s12889-025-22798-x (PMC12020009; doi:10.1186/s12889-025-22798-x)
Supplement: Supplementary file 2 — Supplementary Material 2 [file 12889_2025_22798_MOESM2_ESM.docx]

**Additional file 2 -** **Sensitivity analysis results**

| Table 1. Sensitivity analysis: Comparison of T1 characteristics and T2 outcomes of patients with hypertension, and limited or adequate health literacy | | | | | |  |
| --- | --- | --- | --- | --- | --- | --- |
| **Variables** | **Limited health literacy (n=6,771)** | **Adequate health literacy (n=14,954)** | **Total sample (n=21,725)** | ***p*-value** | **n** | |
| **T1** |  |  |  |  |  | |
| Sex *%* of females | 60.6 | 58.8 | 59.4 | **<0.05^a^** | 21,725 | |
| Age *mean* | 54.2 | 51.6 | 52.4 | **<0.05^b^** | 21,725 | |
| Education *%* |  |  |  |  | 21,149 | |
| Low | 6.6 | 1.6 | 3.2 | **<0.05^a^** |  | |
| Medium | 80.5 | 66.3 | 70.7 |  |  | |
| High | 12.9 | 32.1 | 26.1 |  |  | |
| Monthly income *%* |  |  |  |  | 18,196 | |
| Low | 6.8 | 3.6 | 4.6 | **<0.05^a^** |  | |
| Medium | 71.4 | 59.1 | 62.8 |  |  | |
| High | 21.9 | 37.3 | 32.7 |  |  | |
| Smoking *%* | 15.8 | 14.3 | 14.7 | **<0.05^a^** | 21,412 | |
| Motivation *mean* | 59.9 | 63.6 | 62.5 | **<0.05^b^** | 21,589 | |
| Self-efficacy *mean* | 75.4 | 81.5 | 79.6 | **<0.05^b^** | 21,633 | |
| Problem solving  *mean* | 32.8 | 34.1 | 33.7 | **<0.05^b^** | 21,451 | |
| HRM *%* | 77.3 | 70.4 | 72.6 | **<0.05^a^** | 21,725 | |
| **T2** |  |  |  |  |  | |
| Onset of HRM *%* | 7.5 | 8.4 | 8.1 | **<0.05^a^** | 21,725 | |
| Accumulation of HRM *%* | 5.8 | 4.3 | 4.8 | **<0.05^a^** | 21,725 | |
| Number of illness domains accumulated *%* |  |  |  |  |  | |
| 3 | 4.1 | 3.9 | 4 | **<0.05^b^** | 1,038 | |
| 4 | 0.8 | 0.7 | 0.8 |  |  | |
| 5 | 1.9 | 1.3 | 1.5 |  |  | |
| 6 | 0.3 | 0.1 | 0.2 |  |  | |
| 7 | 0.6 | 0.3 | 0.4 |  |  | |
| 8 | 0.09 | 0.06 | 0.06 |  |  | |
| 9 | 0.1 | 0.04 | 0.07 |  |  | |
| 10 | 0.02 | <0.01 | <0.01 |  |  | |
| Abbreviations: HRM, hypertension-related multimorbidity  Significant *p*-values <0.05 in bold  ^a^ Pearson’s chi-square test  ^b^ Independent sample t-test  All cases from original data | | | | | |  |

| Table 2. Sensitivity analysis: Comparison of T1 prevalence and T2 cumulative incidence of affected illness domains between patients with hypertension, and limited or adequate health literacy | | | | | | | | | |  | |
| --- | --- | --- | --- | --- | --- | --- | --- | --- | --- | --- | --- |
| Illness domain | **T1 HRM prevalence** | | | | **T2 HRM incidence** | | | | | | |
|  | **Limited health literacy**  **(n=6,771)** | **Adequate health literacy (n=14,954)** | ***p*-value** | ***n*** | **Limited health literacy**  **(n=6,771)** | **Adequate health literacy (n=14,954)** | ***p*-value** | ***n*** |  | |  |
| Endocrinology % | 67 | 60.7 | **<0.05** | 11,631 | 13.5 | 11.3 | **<0.05** | 21,725 |  | |  |
| Cardiovascular % | 19 | 14.5 | **<0.05** | 21,502 | 6.7 | 4.9 | **<0.05** | 21,725 |  | |  |
| Haematology % | 1.8 | 1.6 | 0.3 | 21,413 | 0.3 | 0.3 | 0.7 | 20,472 |  | |  |
| Renal ^a^ % | 7.1 | 7.1 | 0.8 | 21,456 | 14.1 | 13.9 | 0.7 | 20,503 |  | |  |
| Respiratory % | 25.3 | 21.6 | **<0.05** | 21,725 | 12.8 | 9 | **<0.05** | 21,725 |  | |  |
| Dermatology % | 1.7 | 2 | 0.1 | 21,725 | 4 | 4.7 | 0.2 | 7,705 |  | |  |
| Psychiatry % | 12.2 | 7.9 | **<0.05** | 21,725 | 8.1 | 6.6 | **<0.05** | 21,725 |  | |  |
| Gastrointestinal % | 60.3 | 52.2 | **<0.05** | 9,916 | 4.8 | 3.1 | **<0.05** | 21,725 |  | |  |
| Neurological % | 13 | 11.8 | **<0.05** | 21,725 | 3.3 | 2.4 | **<0.05** | 21,725 |  | |  |
| Musculoskeletal % | 8.1 | 6.1 | **<0.05** | 21,725 | 15.67 | 10.8 | **<0.05** | 21,725 |  | |  |
| Abbreviations: HRM, hypertension-related multimorbidity  Significant *p*-values <0.05 in bold  Method: Pearson’s chi-square test  ^a^ Renal domain coded without albuminuria  All cases from original data   \| Table 3. Sensitivity analysis: Total effect, natural direct effect and natural indirect effect o*dds ratios* of motivation, self-efficacy beliefs and problem solving, on the association between health literacy and onset and accumulation of hypertension-related multimorbidity \| \| \| \| \| \| \| \| --- \| --- \| --- \| --- \| --- \| --- \| --- \| \| \| \|  \| \| Mediator \| **Onset HRM** \| \| \| **Accumulation of HRM** \| \| \|  \| \| ***TE OR (95%CI)*** \| ***NDE OR (95%CI)*** \| ***NIE OR (95% CI)*** \| ***TE OR (95%CI)*** \| ***NDE OR (95%CI)*** \| ***NIE OR (95% CI)*** \|  \| \| Single mediation \| \| \| \| \| \| \|  \| \| Motivation \| 1.00 (0.99,1.01) \| 1.00(0.99,1.01) \| 1.00 (0.99,1.00) \| **0.99(0.98,0.99)** \| **0.99(0.98,0.99)** \| 0.99(0.99,1.00) \|  \| \| Self-efficacy beliefs \| 1.00(0.99,1.01) \| 1.00(0.99,1.01) \| 1.00(0.99,1.00) \| **0.99(0.98,0.99)** \| **0.99(0.98.0.99)** \| **0.99(0.98,0.99)** \|  \| \| Problem solving \| 1.00(0.99,1.01) \| 1.00(0.99,1.01) \| **0.99(0.98,0.99)** \| **0.99(0.98,0.99)** \| **0.99(0.98.0.99)** \| **0.99(0.98,0.99)** \|  \| \| Joint mediation \| \| \| \| \| \| \|  \| \| M; SEB; PS \| 1.00(0.99,1.01) \| 1.00(0.99,1.01) \| 1.00(0.99,1.00) \| **0.99(0.98,0.99)** \| 0.99(0.98,1.00) \| **0.99(0.98,0.99)** \|  \| \| Abbreviations: TE, total effect; NDE, natural direct effect; NIE, natural indirect effect; HRM, hypertension-related multimorbidity; OR, o*dds ratios,* CI, confidence interval; M, Motivation; SEB, self-efficacy beliefs; PS, problem solving  Significant *confidence intervals* in bold  Method: Natural effect models, adjusted for age, sex, smoking status, education level and monthly income, with multiple imputed data \| \| \| \| \| \| \|  \| | | | | | | | | | |  | |
